# Genetic variations of *β-MYH7* in hypertrophic cardiomyopathy and dilated cardiomyopathy

**DOI:** 10.4103/0971-6866.69348

**Published:** 2010

**Authors:** Reena Tanjore, Advithi RangaRaju, Shivani Vadapalli, Sushant Remersu, Calambur Narsimhan, Pratibha Nallari

**Affiliations:** Department of Genetics, Osmania University, Jamai Osmania P.O., Hyderabad-500 007, India; 1 Kakatiya Medical College, Warangal, Andhra Pradesh, India; 2 CARE Hospitals, Hyderabad, Andhra Pradesh, India

**Keywords:** Diastolic dysfunction, dose effect, dilated cardiomyopathy, hypertrophic cardiomyopathy, single nucleotide polymorphism, systolic dysfunction

## Abstract

**CONTEXT:**

Hypertrophic cardiomyopathy (HCM) is known to be manifested by mutations in 12 sarcomeric genes and dilated cardiomyopathy (DCM) is known to manifest due to cytoskeletal mutations. Studies have revealed that sarcomeric mutations can also lead to DCM. Therefore, in the present study, we have made an attempt to compare and analyze the genetic variations of beta-myosin heavy chain gene (β-MYH7), which are interestingly found to be common in both HCM and DCM. The underlying pathophysiological mechanism leading to two different phenotypes has been discussed in this study. Till date, about 186 and 73 different mutations have been reported in HCM and DCM, respectively, with respect to this gene.

**AIM:**

The screening of β-MYH7 gene in both HCM and DCM has revealed some common genetic variations. The aim of the present study is to understand the pathophysiological mechanism underlying the manifestation of two different phenotypes.

**MATERIALS AND METHODS:**

100 controls, 95 HCM and 97 DCM samples were collected. Genomic DNA was extracted following rapid nonenzymatic method as described by Lahiri and Nurnberger (1991), and the extracted DNA was later subjected to polymerase chain reaction (PCR) based single stranded conformation polymorphism (SSCP) analysis to identify single nucleotide polymorphism (SNP)s/mutations associated with the diseased phenotypes.

**RESULTS AND CONCLUSION:**

Similar variations were observed in β-MYH7 exons 7, 12, 19 and 20 in both HCM and DCM. This could be attributed to impaired energy compromise, or to dose effect of the mutant protein, or to even environmental factors/modifier gene effects wherein an HCM could progress to a DCM phenotype affecting both right and left ventricles, leading to heart failure.

## Introduction

Cardiomyopathy is a disease of the heart muscle, associated with cardiac dysfunction. It is classified as hypertrophic cardiomyopathy (HCM), dilated cardiomyopathy (DCM), restrictive cardiomyopathy (RCM) and arrhythmogenic right ventricular dysplasia (ARVD/C). HCM is characterized by hypertrophy of the left ventricle with the predominant involvement of the interventricular septum (diastolic dysfunction). Its prevalence is found to be 1 in 500.[1,2] DCM is characterized by cardiac dilatation, predominantly of the left ventricle, and weakening of the heart muscle beyond repair, resulting in reduced contractile function and inefficient pumping of the heart (systolic dysfunction). It accounts for 70–80% of all cases of cardiomyopathy.

Till date, about 186 and 73 different mutations have been identified in HCM and DCM, respectively, with respect to *β-MYH7* gene. Since most of the SNPs/ mutations have been reported in this gene, the present study includes the screening of this gene for genetic variations in both the diseases to evaluate the underlying pathophysiological mechanisms leading to either hypertrophy or dilatation.[3,4]

Common genetic variations in exons 7, 12, 19 and 20 of this gene were found in both the HCM and DCM, which emphasizes the role of genetic modifiers and environmental factors along with impaired energy compromise and dosage gene effects of the protein leading to hypertrophy or dilatation eventually resulting in heart failure.

## Materials and Methods

Cardiomyopathies (HCM and DCM) were diagnosed by physical examination, echocardiogram, electrocardiogram and magnetic resonance imaging. Primary DCMs were diagnosed through coronary angiogram, wherein one can rule out the possibility of an underlying myocardial infarction/coronary artery disease, valvular disease and/or other systemic disorders. Blood was collected from 100 healthy blood donors with no history of cardiac disorders and these samples were treated as controls. Blood samples from 97 DCM cases comprising 20 juvenile, 38 primary and 39 secondary cases, and 95 HCM samples were also collected from cardiology units of CARE Hospitals, Mahavir hospitals and Niloufer hospitals, Hyderabad. Informed written consent was obtained from patients in accordance with the study protocol along with the institutional ethics committee’s approval to carry out the above study.

Genomic DNA was extracted by rapid nonenzymatic method as described by Lahiri and Nurnburger.[5] DNA was amplified based on the primer sequences available on Siedman’s database on the website http://genetics.med.harvard.edu/~seidman/cg3/genes/MYH7_exons.html.

Polymerase chain reaction (PCR) was carried out in 0.2 ml tubes containing 100 ng of genomic DNA, 50 pmol each of forward and reverse primers, 0.5–1 U of Taq DNA polymerase enzyme, 200 μM of dNTPs, 1× PCR buffer and water to make up the volume to 25 μl. Initial denaturation was carried out at 95°C for 3 min, followed by a denaturation step at 95°C for 30 s. The annealing temperature varied from 54°C to 65°C based on the exon for 30 s and an extension at 72°C for 1 min. A final extension step of 72°C was for 2 min at the end of the reaction.

The amplified DNA samples were subjected to SSCP analysis on nondenaturing/polyacryalamide gels. Samples revealing any kind of band pattern variation or mobility shift were commercially sequenced on a 3730 × l DNA analyzer (Macrogen, Seoul Korea).

## Results

Screening of MYH7 gene in HCM and DCM patients revealed common genetic variations in exons 7, 12, 19 and 20. Apart from these, unique SNPs/mutations in HCM (NCBI accession no. 51855930–51855950) and DCM (NCBI accession no. EF630363–EF630367 and EU091311–EU091316) were found [Reena *et al*. 2006,[6] Boda *et al*. 2009 (communicated)]. The list of SNPs/ mutations which are found to be common in both the diseased types is given in Table 1.[6]

**Table 1:** Mutations/SNPs observed in both HCM and DCM

Exon	Common variations		Control (n)	Patient (n)	NCBI accession no. in DCM
	HCM	DCM			
7	Ala199Ala nt (7647) heterozygous	Ala199Ala nt (7647) homozygous	—	One patient each in both HCM and DCM	EF630363
12	Gly354Gly (9600) heterozygous	Gly354Gly(9600) homozygous	1	Six in HCM and two in DCM	EF630364
	Lys365Lys(9633) heterozygous	Lys365Lys(9633) homozygous	—	One in HCM and three in DCM	EF630365
19	IVS19 + 92A/G homozygous	IVS19 + 82A/G homozygous	—	One each in HCM and DCM	EU091312
20	IVS19 − 56A/G homozygous	IVS19 − 56A/G homozygous	—	One in HCM and two in DCM	EU091314

**Figure 1:**
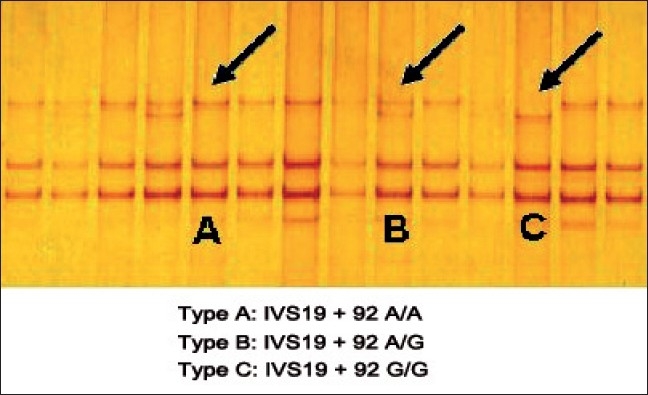
SSCP analysis of exon 19 in HCM

**Figure 2:**
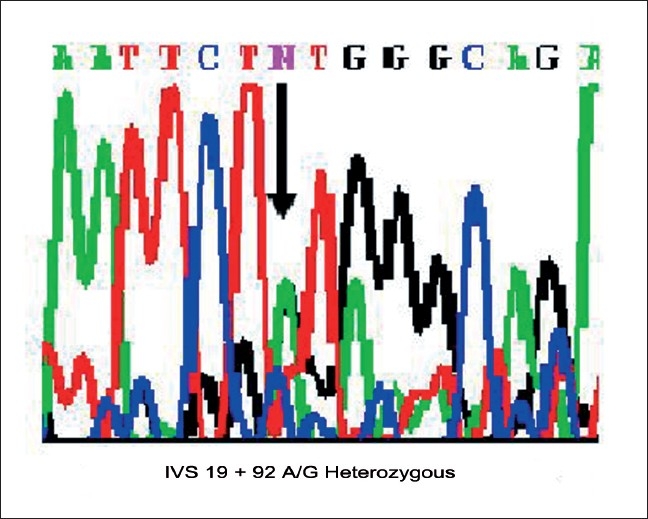
Chromatogram showing the intronic variation in Exon 19 in HCM

**Figure 3:**
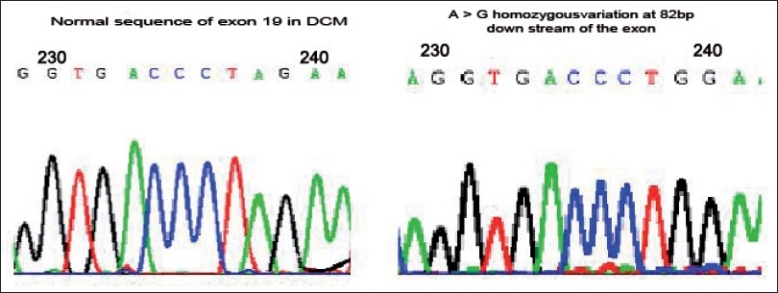
Fig 1b: Normal sequence of 82 bases downstream of the exon 19 in DCM; Fig 1c: A>G homozygous variation at 82 bases downstream of the exon 19 in DCM

**Figure 4:**
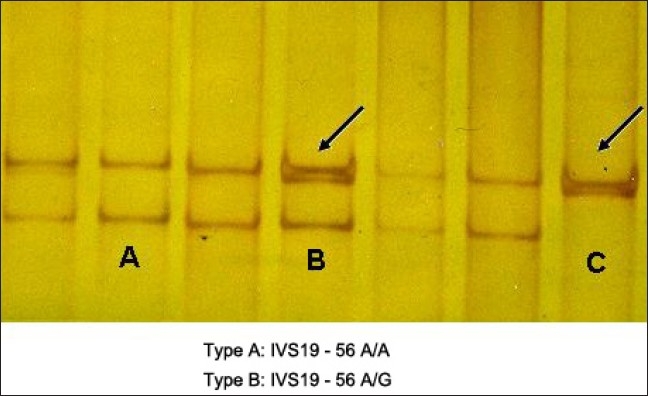
SSCP analysis of exon 20 in HCM

Mutational screening of *MYH7* gene in HCM revealed five SNPs in exons 7, 12, 19 and 20 of which three were heterozygous and two were homozygous, [Figure 1, 2, 4] whereas the same SNPs were found to be homozygous [Figure 2] in DCM [Figure 3] sample, revealing the dose effect of the protein with the gross anatomical variations in the ventricles leading to heart failure in DCM cases.

## Discussion

Myosin is the most conserved and a major contractile protein, which helps in force generation in the myocardium. Since the maximum number of mutations or SNPs are found to be in *MYH7*, we made an attempt to screen this gene in both HCM and DCM. Although we could find distinct SNPs/mutations in HCM and DCM, interestingly, certain common genetic variations were found in exons 7, 12, 19 and 20 of *MYH7* gene in both the diseased phenotypes, where three of the genotypes were heterozygotes in HCM and all the genotypes were homozygotes in DCM. In the present study, we tried to hypothesize the underlying pathophysiological mechanism where the same SNPs/mutations in one gene would lead to two different diseased phenotypes.

There are various hypotheses explaining the underlying pathophysiological mechanism.

### Dose effect

Dose effect of the mutant protein plays a role, where the heterozygous condition leads to hypertrophy and homozygous [Figure 4] condition leads to DCM phenotype resulting in heart failure. This is further supported by a previous study in which heterozygous mice expressing Arg403Gln *α-MHC* developed left ventricular hypertrophy as commonly seen in HCM, whereas homozygous mice developed progressive DCM leading to neonatal death.[7–9]

### Phenotypic plasticity

Genotype-phenotype correlations can be best explained by phenotypic plasticity where mutations/variations in the same gene and even the same mutation/variation in a different background cause disparate phenotypes.[10] In the case of cardiomyopathies, such phenotypic plasticity was best illustrated by Dr. Seidman’s group, who reported that mutations in the *MYH7* and *TNNT2*, encoding beta-myosin heavy chain and cardiac troponin T, respectively, could cause either HCM or DCM, the opposite ends of the spectrum of phenotypic responses of the heart to injury, stress or mutations.[11]

### Alterations in myofibrillar Ca^2+^

Same variations leading to different diseased phenotypes can also be explained by the energy compromise and alterations in myofibrillar Ca^2+^ sensitivity pathways. Impaired myofibrillar Ca^2+^ sensitivity and force transmission of cytoskeletal protein variations could result in ventricular dilatation in DCM. On the other hand, sensitization to Ca^2+^ is likely to promote systolic function in HCM but impaires diastolic function due to an increase in cytosolic free Ca^2+^.[12,13] A study on Indians by *Taranjit Singh et al*.[14] with a similar approach, in which *MYH7* mutations were found in both HCM and DCM, further supports our data and reconfirms the fact indicating that there is a wide genetic and phenotypic heterogeneity of HCM and DCM in our population similar to that reported for other ethnic populations such as Caucasians and Japanese.[15–20]

In addition to the above discussed reasons, environmental factors or modifier gene effect could also be playing a significant role in diverse phenotypes. Therefore, our results have reinforced the fact that the progression of hypertrophy to dilatation could finally lead to heart failure.

### Genotype beta–phenotype correlation

Gross abnormalities in *β-MYH7* might have a little pathogenic potential in the heterozygous state and minor abnormalities may have a causative role proving dominant negative effect of the mutated protein. Polymorphisms that are associated with the disease condition could be present in the regulatory region of the gene and potentially affect the expression of the genes and hence may be relevant to disease susceptibility. Heterozygous condition is associated with mild hypertrophy (with or without obstruction), relatively good prognosis, agedependent penetrance and longer life span, whereas the homozygous state is associated with an increased risk of the disease at an early age. The heterozygous SNPs are regulatory in nature acting as susceptiblity alleles. Early onset of the disease is predicted in homozygous state with predominant left ventricle and left auricle dilatations with Ejection Fraction (EF) (>40%) along with global hypokinesia in DCM. Hypertrophy in homozygous condition may progress to DCM condition and eventually leads to heart failure.

The clinical utility of such findings can be correlated to poor prognosis and sudden cardiac deaths in DCM compared to HCM, based on the dosage effect. A report of Reena *et al*.[6] on genetic testing of *β-MYH7* in a large kindred helped in the prediction of asymptomatic, presymptomatic and symptomatic individuals based on the mutation R870H and identified the genotype phenotype correlation being established.[6] SNPs/ mutations have been detected in other sarcomeric genes in patients not harboring *β-MYH7*, revealing genetic heterogeneity of the condition.

## Conclusion

In the present study, common genetic variations were found in *MYH7* gene in both HCM and DCM samples This could be attributed to impaired energy compromise, or to dose effect of the mutant protein, or to even environmental factors/modifier gene effects responsible for an HCM to progress to a DCM phenotype in which both right and left ventricles are affected culminating into heart failure.
